# Leakage Prediction in Machine Learning Models When Using Data from Sports Wearable Sensors

**DOI:** 10.1155/2022/5314671

**Published:** 2022-05-17

**Authors:** Qizheng Dong

**Affiliations:** Zhengzhou University of Science and Technology, Zhengzhou, Henan 450000, China

## Abstract

One of the major problems in machine learning is data leakage, which can be directly related to adversarial type attacks, raising serious concerns about the validity and reliability of artificial intelligence. Data leakage occurs when the independent variables used to teach the machine learning algorithm include either the dependent variable itself or a variable that contains clear information that the model is trying to predict. This data leakage results in unreliable and poor predictive results after the development and use of the model. It prevents the model from generalizing, which is required in a machine learning problem and thus causes false assumptions about its performance. To have a solid and generalized forecasting model, which will be able to produce remarkable forecasting results, we must pay great attention to detecting and preventing data leakage. This study presents an innovative system of leakage prediction in machine learning models, which is based on Bayesian inference to produce a thorough approach to calculating the reverse probability of unseen variables in order to make statistical conclusions about the relevant correlated variables and to calculate accordingly a lower limit on the marginal likelihood of the observed variables being derived from some coupling method. The main notion is that a higher marginal probability for a set of variables suggests a better fit of the data and thus a greater likelihood of a data leak in the model. The methodology is evaluated in a specialized dataset derived from sports wearable sensors.

## 1. Introduction

Machine learning models typically receive input data and solve problems such as pattern recognition by applying a sequence of particular transformations. The majority of these transformations turn out to be extremely sensitive to modest changes in input. Under specific scenarios, using this sensitivity can result in a difference in the behavior of the learning algorithm [1, 2]. Adversarial attack is the design of an adequate input in a specific way that leads the learning algorithm to erroneous outputs while not easily noticed by human observers. It is a severe concern in the reliability and security of artificial intelligence technologies. The issue arises because learning techniques are intended for use in stable situations where training and test data are generated from the same, possibly unknown distribution [3]. A trained neural network, for example, represents a significant decision limit corresponding to a standard class. Of course, the restriction is not without flaws. A correctly designed and implemented attack, which corresponds to a modified input form a slightly differentiated dataset, can cause the algorithm to make an incorrect judgment (wrong class) [4–6].

Developing and selecting machine learning methodologies to solve complex, usually nonlinear, problems is inextricably linked to the area of application and the target problem it seeks to solve. This is one of the essential processes of preprocessing the area of interest and the dataset, as the choice of appropriate algorithms depends on not only the nature and dynamics of the problem but also the characteristics of the available data, such as volume, number, and type of variables in question. The preprocessing of the data concerns the tests and the preparation work that should be carried out in the examined dataset before the use and application of machine learning algorithms. This method is critical because if the quality of usage or training data is not ensured, the algorithms' performance will be subpar or the algorithms may produce false results [6, 7].

In general, data preparation/preprocessing entails dealing with scenarios when the original data have issues such as contradicting information, coding discrepancies, field terminology, and units of measurement. However, more critical issues such as the presence of lost values, noise, and extreme values and dealing with special requirements that necessitate data transformation, such as discretization, normalization, dimension reduction, or the selection of the most appropriate features, must be addressed [9–11]. It should be noted that several techniques can be used in preprocessing processes, with the choice of the best strategy arising from the nature of the field of knowledge, the problem to be addressed, the available data, and the machine learning algorithm used.

One of the most critical errors that occur during the preprocessing of data for use by machine learning algorithms is data leakage. The leak in question refers to cases where, inadvertently or even intentionally, the value that the model wishes to predict (dependent variable) is contained indirectly or directly in the features that are called to train the algorithm (independent variables). Any variable that provides transparent information about the value that the model is trying to predict is considered a data leak and leads to fictitious results. An obvious solution to this problem is to apply preprocessing only to the training set. Using preprocessing techniques to the whole dataset will make the model learn the training and the test sets, resulting in a data leak, and thus the model fails to generalize [2, 12, 13].

The major problem of data leakage occurs when there is a severe indirect interaction of features which is not easy to detect. It is, for example, a widespread phenomenon in machine learning experiments; the relationship between the dependent and the independent variable is complex (e.g., polynomial, trigonometric, and so on), so new features may be created that seem to help capture this relationship. Still, in practice, they create serious data leaks [14, 15].

Similarly, combinations may exist between independent and dependent variables through, for example, an arithmetic operation, a modification, or a conversion to make them more important in explaining the discrepancies in the data than if they remained separate. Creating a new opportunity through the interaction of existing features creates data leaks and significant bias in the final machine learning model [4, 7, 11].

For example, Lu et al. [15] developed a weighted context graph model (WCGM) for information leakage, with the critical goals of first increasing the contextual relevance of information, second classifying the tested data based on the commonality characteristics of its context graphs, and third preserving data proprietors' privacy. The weighted context network reduces complexity by using key sensitive phrases as nodes and contextual linkages as edges. The proposed maximum subgraph matching approach and deep learning algorithms are used to evaluate the similarity of the tested information and the pattern, as well as the responsiveness of the tested data to match the converted data better. The proposed model surpassed the competition regarding accuracy, recall, and run time, indicating its ability to detect real-time data leaks.

Using a variety of datasets, Salem et al. [14] provided research on the new and developing danger of membership inference attacks, demonstrating the efficacy of the suggested assaults across sectors. They offer two defensive strategies to alleviate the problem. The first, known as dropout, involves randomly deleting specific nodes in each fully linked neural system training step. In contrast, the second, known as model stacking, involves organizing numerous ML models in a ranked order [16]. Extensive testing has shown that our defensive strategies may significantly lower the performance of a membership inference attempt while retaining a high degree of usefulness, i.e., good target model prediction accuracy. They also suggest a defensive mechanism against a larger class of inclusion inference assaults while maintaining the ML model's high usefulness.

In this work, we proposed an innovative system of leakage prediction in machine learning models, which calculates a lower limit for the marginal probability of the observed variables coming from a coupling method, which shows that in an examined machine learning model, there is data leakage. The methodology is implemented based on the Bayesian inference methodology [17–19]. The model's goal is to generate an analytical approach to the reverse probability of unobserved variables [20, 21], to draw statistical inferences about the important correlated variables, and to compute a lower limit for the marginal likelihood of observable variables generated from a coupling method. The highest probability indicates that there is a data leak [22]. This is done to have a solid and generalized forecasting model, which will produce remarkable forecasting results without data leakages.

## 2. Proposed Approach

**Figure 1:**
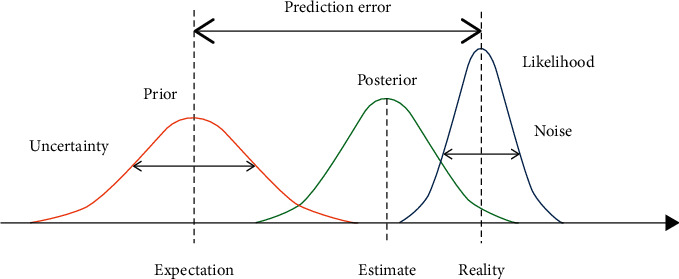
Bayesian inference.

The proposed implementation is based on Bayesian inference [23–25], which is a method of approaching intractable problems that arise in highly fuzzy environments. More specifically, the methodology offers a secure solution for the observed variables and unknown parameters and latent states of variables, characterized by different types of relationships (interconnected, transformed, hidden, random, and so on). A prior distribution, a posterior distribution, and a likelihood function are used to illustrate Bayesian inference [26] in Figure 1.

The prediction error is defined as the difference between the previous expectation and the likelihood function's peak (i.e., reality). The variance of the prior is the source of uncertainty. The variance of the likelihood function is referred to as noise [27].

Parameters and latent variables are grouped as “unobserved variables.” So, with the proposed method, the purpose is as follows [28–31]:

1. In order to generate an analytical approach to the reverse probability of unobserved variables, develop statistical findings for the important correlated variables.
2. The marginal likelihood of the data presented in the model can be used to derive a lower limit for the marginal probability of the observed data, with the marginalization conducted on unobserved variables. The main notion is that a higher marginal probability for a set of variables suggests a better fit of the data and thus a greater likelihood of a data leak in the model.

**Figure 2:**
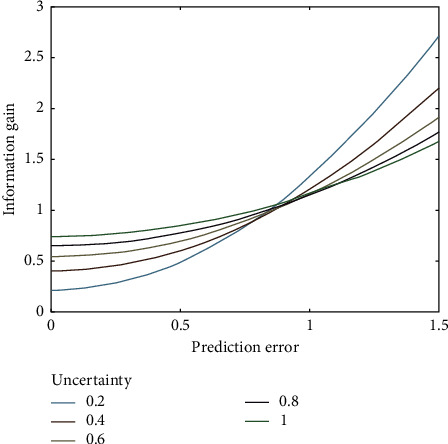
Information gain vs prediction error.

An example of information gain vs prediction error is presented in Figure 2.

Information gain is calculated mathematically as a function of prediction errors for uncertainty levels ranging from 0.2 to 1.0. The external noise level is set to 0.1 [23, 27].

The method generally approaches a conditional latent variable density given the observed variables where we assume that a mixture is present. Mixing behavior occurs because the source of each observation is unknown, that is, the classification into a specific, exact domain of a variable [32]. Thus, each observation xi is predetermined to each of *f*_*i*_(·∣*θ*_*i*_) with probability pi. Depending on the case, the purpose of the inference is to reconstruct the classification of observations into definition fields, construct estimators for the components' parameters, or even estimate the number of components themselves [15]. It is always feasible to map a mixture of *k* form distributions to a random variable *Xi* via a delimitation method [25, 33]:

(1) $$\begin{array}{c} \sum_{I=1}^{K}p_{i}f_{i}\left(x|\theta_{i}\right). \end{array}$$

The random variable *Zi* with {1, 2,…, *k*}, is as follows [34]:

(2) $$\begin{array}{c} X_{i}|Z_{i}=z∼f\left(x|\theta_{z}\right)\mu \varepsilon Z_{i}∼M_{k}\left(1;p_{1},\ldots ,p_{k}\right). \end{array}$$

Next, we assume that we have observed the extended data, which consist of independent pairs with distribution [35]:

(3) $$\begin{array}{c} P\left(Z_{i}=j|X_{i}=x\right)=\frac{p_{j}f_{j}\left(x\right)}{\sum_{I=1}^{K}p_{i}f_{i}\left(x\right)}\propto p_{j}f_{j}\left(x\right). \end{array}$$

In the particular case of the model:

(4) $$\begin{array}{c} pN\left(\mu_{1},1\right)+\left(1-p\right)N\left(\mu_{2},1\right), \end{array}$$

where we consider the same normal a priori distribution in the media, *μ*_1_, *μ*_2_ ~ *N*(0,10), we will calculate the ex post weight *ω*(*z*) for a classification *z*, where in the first component are *l* observations [24, 36]:

(5) $$\begin{array}{c} \sum_{I=1}^{N}I_{\left\{z_{t}=1\right\}}=l\text{ for}\left(n_{1},n_{2}\right)=\left(l,n-l\right). \end{array}$$

So, we have [37]

(6) $$\begin{array}{c} \pi \left(\underline{z},\mu_{1},\mu_{2}|\underline{x},n_{1},n_{2}\right)\propto \;\exp\left\{-\frac{1}{2}\sum_{i=1}^{n}\left[I_{\left\{z_{i}=1\right\}}{\left(x_{i}-\mu_{1}\right)}^{2}+\left(1-I_{\left\{z_{t}=1\right\}}\right){\left(x_{i}-\mu_{2}\right)}^{2}\right]-\frac{\mu_{1}^{2}}{20}-\frac{\mu_{2}^{2}}{20}\right\}\times \frac{1}{{\left(2\pi \right)}^{n/2}}p^{\sum_{i=1}^{n}I_{\left\{z_{i}-1\right\}}}{\left(1-p\right)}^{n-\sum_{i=1}^{n}I_{\left\{z_{i}-1\right\}}}. \end{array}$$

The ex-weight *ω*(*z*) is obtained by completing the above function in *RxR* for *μ*1 and *μ*2, which is a double integral which is easily calculated. For the completion in terms of *μ*1, excluding the parts that do not contain it, it is enough to calculate [24, 33, 36, 38]

(7) $$\begin{array}{c} I_{1}=\int_{-\infty }^{+\infty }\exp\left\{-\frac{1}{2}\sum_{i=1}^{n}I_{z_{l}=1}{\left(x_{i}-\mu_{1}\right)}^{2}-\frac{\mu_{1}^{2}}{20}\right\}d\mu_{1}. \end{array}$$

But

(8) $$\begin{array}{c} \exp\left\{-\frac{1}{2}\sum_{i=1}^{n}I_{\left\{z_{i}=1\right\}}{\left(x_{i}-\mu_{1}\right)}^{2}-\frac{\mu_{1}^{2}}{20}\right\}\\ =\exp\left\{-\frac{1}{2}\mu_{1}^{2}\left(\sum_{i=1}^{n}I_{\left\{z_{i}=1\right\}}+\frac{1}{10}\right)-\frac{1}{2}\sum_{i=1}^{n}x_{i}^{2}I_{\left\{z_{i}=1\right\}}+\mu_{1}\sum_{i=1}^{n}x_{i}I_{\left\{z_{i}=1\right\}}\right\}\\ =\exp\left\{-\frac{1}{2}\left(\sum_{i=1}^{n}I_{\left\{z_{i}=1\right\}}+\frac{1}{10}\right)\left(\mu_{1}^{2}+\frac{\sum_{i=1}^{n}x_{i}^{2}I_{\left\{z_{i}=1\right\}}}{\sum_{i=1}^{n}I_{\left\{z_{i}=1\right\}}+1/10}-2\mu_{1}\frac{\sum_{i=1}^{n}x_{i}I_{\left\{z_{i}=1\right\}}}{\sum_{i=1}^{n}I_{\left\{z_{i}=1\right\}}+1/10}\right)\right\}\\ =\exp\left\{\frac{1}{2}\left(\sum_{i=1}^{n}I_{\left\{z_{i}=1\right\}}+\frac{1}{10}\right){\left(\frac{\sum_{i=1}^{n}x_{i}I_{\left\{z_{i}=1\right\}}}{\sum_{i=1}^{n}I_{\left\{z_{i}=1\right\}}+1/10}\right)}^{2}\right\}\\ \times \;\exp\left\{-\frac{1}{2}\left(\sum_{i=1}^{n}I_{\left\{z_{i}=1\right\}}+\frac{1}{10}\right)\left(\mu_{1}^{2}+\frac{\sum_{i=1}^{n}x_{i}^{2}I_{\left\{z_{i}=1\right\}}}{\sum_{i=1}^{n}I_{\left\{z_{i}=1\right\}}+1/10}-2\mu_{1}\frac{\sum_{i=1}^{n}x_{i}I_{\left\{z_{i}=1\right\}}}{\sum_{i=1}^{n}I_{\left\{z_{i}=1\right\}}+1/10}+{\left(\frac{\sum_{i=1}^{n}x_{i}I_{\left\{z_{i}=1\right\}}}{\sum_{i=1}^{n}I_{\left\{z_{i}=1\right\}}+1/10}\right)}^{2}\right)\right\}\\ \left.=c_{1}\exp\left\{-\frac{1}{2}\right.\left(\sum_{i=1}^{n}I_{izz},=1+\frac{1}{10}\right)\left(\mu_{1}^{2}-2\mu_{1}\frac{\sum_{i=1}^{n}x_{i}I_{\left\{z_{i}=1\right\}}}{\sum_{i=1}^{n}I_{\left\{z_{i}=1\right\}}+1/10}+{\left(\frac{\sum_{i=1}^{n}x_{i}I_{\left\{z_{i}=1\right\}}}{\sum_{i=1}^{n}I_{\left\{z_{i}=1\right\}}+1/10}\right)}^{2}\right)\right\}\\ =c_{1}\exp\left\{-\frac{1}{2}\left(\sum_{i=1}^{n}I_{\left\{z_{i}=1\right\}}+\frac{1}{10}\right){\left(\mu_{1}-\frac{\sum_{i=1}^{n}x_{i}I_{\left\{z_{i}=1\right\}}}{\sum_{i=1}^{n}I_{\left\{z_{i}=1\right\}}+1/10}\right)}^{2}\right\},\\ \text{where }c_{1}=\exp\left\{-\frac{1}{2}\sum_{i=1}^{n}x_{i}^{2}I_{\left\{z_{i}=1\right\}}+\frac{{\left(\sum_{i=1}^{n}x_{i}I_{\left\{z_{i}=1\right\}}\right)}^{2}}{2\left(l+1/10\right)}\right\}, \end{array}$$

So, to calculate the integral, we have

(9) $$\begin{array}{c} I_{1}=c_{1}\int_{-\infty }^{+\infty }\exp\left\{-\frac{1}{2}\left(\sum_{i=1}^{n}I_{\left\{z_{t}=1\right\}}+\frac{1}{10}\right){\left(\mu_{1}-\frac{\sum_{i=1}^{n}x_{i}I_{\left\{z_{t}=1\right\}}}{\sum_{i=1}^{n}I_{\left\{z_{t}=\right\}1}+1/10}\right)}^{2}\right\}d\mu_{1}\Rightarrow \\ I_{1}=c_{1}\frac{\sqrt{2\pi }}{\sqrt{\sum_{i=1}^{n}I_{\left\{z_{t}=1\right\}}+1/10}}=c_{1}\frac{\sqrt{2\pi }}{\sqrt{l+1/10}}. \end{array}$$

because the last integral is crucial in the full support of the exponential distribution [39]:

(10) $$\begin{array}{c} N\left(\frac{\sum_{i=1}^{n}x_{i}I_{\left\{z_{t}=1\right\}}}{\sum_{i=1}^{n}I_{\left\{z_{t}=1\right\}}+1/10},\frac{1}{\sum_{i=1}^{n}I_{\left\{z_{t}=1\right\}}+1/10}\right). \end{array}$$

For the completion in terms of *μ*2, excluding the parts that do not contain it, it is enough to calculate [23, 36, 38, 40]

(11) $$\begin{array}{c} I_{2}=\int_{-\infty }^{+\infty }\exp\left\{-\frac{1}{2}\sum_{i=1}^{n}\left(1-I_{\left\{z_{t}=1\right\}}\right){\left(x_{i}-\mu_{2}\right)}^{2}-\frac{\mu_{2}^{2}}{20}\right\}\text{d}\mu_{2}. \end{array}$$

Following the same methodology as before, we conclude that [41]

(12) $$\begin{array}{c} I_{1}=c_{2}\frac{\sqrt{2\pi }}{\sqrt{n-l+1/10}}\\ \text{where }c_{2}=\exp\left\{-\frac{1}{2}\sum_{i=1}^{n}x_{i}^{2}\left(1-I_{\left\{z_{t}=1\right\}}\right)+\frac{{\left(\sum_{i=1}^{n}x_{i}\left(1-I_{\left\{z_{i}=1\right\}}\right)\right)}^{2}}{2\left(n-l+1/10\right)}\right\}. \end{array}$$

So, the ex post probability *ω*(*z*) is calculated as follows [21, 23, 42, 43]:

(13) $$\begin{array}{c} \omega \left(\underline{z}\right)=c_{1}\frac{\sqrt{2\pi }}{\sqrt{l+1/10}c_{2}\sqrt{2\pi }/\sqrt{n-l+1/10}}p^{\sum_{i=1}^{n}I_{\left[z-1\right]}-1}{\left(1-p\right)}^{\left.n-\sum_{i=1}^{n}I_{zz}-1\right\}}\\ =c_{1}c_{2}\frac{2\pi }{\sqrt{\left(l+1/10\right)\left(n-l+1/10\right)}}p^{l}{\left(1-p\right)}^{n-l}. \end{array}$$

If we replace *c*1, *c*2, we take the relation:

(14) $$\begin{array}{c} \omega \left(\underline{z}\right)=\frac{\sqrt{2\pi }}{\sqrt{\left(l+1/10\right)}\sqrt{\left(n-l+1/10\right)}}\times \;\exp\left\{-\frac{1}{2}\left(\sum_{i=1}^{n}x_{i}^{2}-\frac{{\left(\sum_{i=1}^{n}x_{i}I_{\left\{z_{t}=1\right\}}\right)}^{2}}{l+1/10}-\frac{\left.{\left(\sum_{i=1}^{n}x_{i}\left(1-I_{\left\{z_{t}=1\right\}}\right)\right)}^{2}\right)}{\left.n-l+1/10\right)}\right)p^{l}{\left(1-p\right)}^{n-l}.\right. \end{array}$$

Thus, from the above analysis, it appears that it is practically possible to arrive at detailed expressions of the maximum probability and Bayes estimators [44] for the ex ante distributions of the variables of interest and thus marginalize the set of variables for models where there is a data leak [28, 33].

## 3. Experiments and Results

A specialized scenario was implemented to model the proposed system that uses sports wearables data to record the movements of athletes playing beach volleyball. The dataset comprises three-dimensional acceleration data from joint actions of beach volleyball athletes, each of whom was fitted with an accelerometer worn on the wrist and sampled at 39 Hz. The signal was recorded at 14 bits per axis and then compressed to 16 g. The *x*, *y*, and *z* axes relate to the athletes' spatial arrangement, which is recorded in an independent coordinate system based on the sensor configuration, as there was no transfer to real-world coordinates [45, 46]. The 30 athletes recorded ranged in expertise from novice to professional volleyball players. The set's goal is to create an identification and classification system that extracts relevant portions from continuous input and classifies them [47]. The categorization includes ten various volleyball activities, such as homemade service, block, nail, and so on. For the evaluation of the system, 10 characteristics were selected, which were randomly combined into pairs to identify the observed variables, whether they come from a coupling method and whether there is a data leak.

We first describe some key features. Let *g*(·, ·*|θ*) be the joint density function of (*X*, *Z*) given by the parametric vector *θ*, *f*(·*|θ*) be the density function of *X* given *θ*, and *k*(·*|x*, *θ*) be the function density of the bounded distribution of *Z* given by observations *x* and *θ*. The algorithm is based on the use of incomplete data, i.e., we can write the distribution of sample *x* as follows [1, 2, 40]:

(15) $$\begin{array}{c} f\left(x|\theta \right)=\int g\left(\underline{x},\underline{z}|\theta \right)\text{d}\underline{z}\\ =\int f\left(x|\theta \right)k\left(\underline{z}|\underline{x},\theta \right)\text{d}\underline{z}. \end{array}$$

So, logarithm it:

(16) $$\begin{array}{c} g\left(x,z|\theta \right)=f\left(x|\theta \right)k\left(\underline{z}|\underline{x},\theta \right). \end{array}$$

We arrive at a complete (unobserved) logarithm of probability:

(17) $$\begin{array}{c} L^{c}\left(\theta |\underline{x},\underline{z}\right)=L\left(\theta |\underline{x}\right)+\text{log}k\left(\underline{z}|\underline{x},\theta \right), \end{array}$$

where *L* is the observed logarithm of the probability. The algorithm fills in the missing variables *z* based on *k* (*z*|*x*, *θ*) and then maximizes with *θ* the expected full logarithm probability [21, 25, 48].

So, the algorithm is configured as follows:

1. )Give some initial values to *θ*(0).
2. )For each *t*, *t* = 1, 2,…, *n*, calculate $Q\left(\theta |\theta^{\left(t-1\right)},\underline{x}\right)=E_{\theta^{\left(t-1\right)}}\left(L^{c}\left(\theta |\underline{x},\underline{Z}\right)\right)$ where $\underline{Z}∼k\left(z|x,\theta \right)$.
3. )Maximize concerning *θ* the $Q\left(\theta |\theta^{\left(t-1\right)},\underline{x}\right)$ and set $\theta^{\left(t\right)}=\arg\underset{\theta }{\max}Q\left(\theta |\theta^{\left(t-1\right)},\underline{x}\right)$.

When performing the above algorithm, the result is that in each iteration, the (observed) *L*(*θ*|*x*) increases.

As an application of the above, we consider the particular case of the model of mixing two regular variables, where all parameters are known except *θ* = (*μ*1, *μ*2). For a simulated sample of 500 observations and actual values *p*=0.7 and (*μ*1, *μ*2) = (0, 2.5), the logarithm of probability has two peaks. Applying the algorithm to this model, we have that the total probability is [20, 49, 50]

(18) $$\begin{array}{c} p^{\sum_{i=1}^{n}I_{izi}-1}{\left(1-p\right)}^{n-\sum_{i=1}^{n}I\left.z_{i}-1\right\}}{\left(2\pi \right)}^{-n/2}\exp\left\{-\frac{1}{2}\sum_{i=1}^{n}\left[I_{\left\{z_{t}=1\right\}}{\left(x_{i}-\mu_{1}\right)}^{2}+\left(1-I_{\left\{z_{t}=1\right\}}\right){\left(x_{i}-\mu_{2}\right)}^{2}\right]\right\}, \end{array}$$

where its logarithm is

(19) $$\begin{array}{c} L^{c}\left(\theta \underline{x},\underline{z}\right)=\sum_{i=1}^{n}I_{\left\{z_{t}=1\right\}}\log\;p+\left(n-\sum_{i=1}^{n}I_{\left\{z_{t}=1\right\}}\right)\log\left(1-p\right)-\frac{n}{2}\log\left(2\pi \right)-\frac{1}{2}\sum_{i=1}^{n}\left[I_{\left\{z_{t}=1\right\}}{\left(x_{i}-\mu_{1}\right)}^{2}+\left(1-I_{\left\{z_{t}=1\right\}}\right){\left(x_{i}-\mu_{2}\right)}^{2}\right]. \end{array}$$

For the first step, we need to calculate

(20) $$\begin{array}{c} Q\left(\theta |\theta^{\left(t-1\right)},\underline{x}\right)=E_{\theta^{\left(t-1\right)}}\left(\text{log}L^{c}\left(\theta |\underline{x},\underline{Z}\right)\right), \end{array}$$

where the mean value is taken for $\underline{Z}∼k\left(z|x,\theta \right)$, and we have that *Zi* are independent of [51–54]

(21) $$\begin{array}{c} P\left(Z_{i}=1|\underline{\theta },\underline{x}\right)=\frac{p\text{exp}\left\{-{\left(x_{i}-\mu_{1}\right)}^{2}/2\right\}}{p\text{exp}\left\{-{\left(x_{i}-\mu_{1}\right)}^{2}/2\right\}+\left(1-p\right)\text{exp}\left\{-{\left(x_{i}-\mu_{2}\right)}^{2}/2\right\}}=1-P\left(Z_{i}=2|\underline{\theta },\underline{x}\right). \end{array}$$

In step *t*, the expected rankings are equal to

(22) $$\begin{array}{c} {\hat{z}}_{i}^{\left(t-1\right)}=E\left(\sum_{i=1}^{n}I_{\left\{z_{t}=1\right\}}|{\underline{\theta }}^{\left(t-1\right)},\underline{x}\right)\\ =P\left(Z_{i}=1|{\underline{\theta }}^{\left(t-1\right)},\underline{x}\right). \end{array}$$

Therefore:

(23) $$\begin{array}{c} Q\left(\theta |\theta^{\left(t-1\right)},\underline{x}\right)=\overset{n}{\underset{i=1}{\sum }}{\hat{z}}_{i}^{\left(t-1\right)}\log\;p+\left(n-\sum_{i=1}^{n}{\hat{z}}_{i}^{\left(t-1\right)}\right)\log\left(1-p\right)-\frac{n}{2}\log\left(2\pi \right)\\ -\frac{1}{2}\overset{n}{\underset{i=1}{\sum }}\left[{\hat{z}}_{i}^{\left(t-1\right)}{\left(x_{i}-\mu_{1}\right)}^{2}+\left(1-{\hat{z}}_{i}^{\left(t-1\right)}\right){\left(x_{i}-\mu_{2}\right)}^{2}\right]. \end{array}$$

which we maximize in the second step in terms of (*μ*1, *μ*2) and get

(24) $$\begin{array}{c} \mu_{1}^{\left(t\right)}=\frac{\sum_{i=1}^{n}{\hat{z}}_{i}^{\left(t-1\right)}x_{i}}{\sum_{i=1}^{n}{\hat{z}}_{i}^{\left(t-1\right)}},\\ \mu_{2}^{\left(t\right)}=\frac{\sum_{i=1}^{n}1-{\hat{z}}_{i}^{\left(t-1\right)}x_{i}}{\sum_{i=1}^{n}1-{\hat{z}}_{i}^{\left(t-1\right)}}. \end{array}$$

This example involved running the algorithm 20 times (each time with 100 repeats) while picking random numbers from a range of possibilities for the initial conditions. However, the proposed approach was only drawn to the highest and principal vertex of the logarithm probability eight times out of every 20 times in the experiments. It was drawn to the pseudo-vertex of the logarithm probability distribution for the remaining 12 times (although the likelihood is much lower). The original values were closer to the lower peak than the final values, indicating that the early values were more accurate. The algorithm converges to the pseudo-peak of likelihood, at which point we may make 84 percent correct predictions about the coupling between the variables in the dataset. Accordingly, we will have 93 percent of the variables accurately predicted to couple their coefficients if the algorithm converges to the dominant peak in probability.

## 4. Discussion and Conclusions

In this work, we proposed an innovative system of leakage prediction in machine learning models, which is based on Bayesian inference, to calculate a lower limit for the marginal probability of the observed variables coming from a coupling method, which shows that in an examined machine learning model, there is data leakage. The methodology is evaluated in a specialized dataset from sports wearable sensors, where the ability of the method to detect variable coupling is demonstrated, even when it is done randomly.

The proposed methodology is a Bayesian approach to statistical discoveries in complicated distributions that are difficult to evaluate directly or by sampling, and this is the methodology that has been offered. It is a method of selection that is different from Monte Carlo sampling methods. While Monte Carlo techniques use a sequence of samples to approximate a rear distribution numerically, the proposed algorithm provides a locally optimal, correct analytical solution, allowing even hidden variable coupling to be found. From the maximum ex post estimate of each variable's unique most probable value to the fully Bayesian estimation that calculates (approximately) the entire rear distribution of parameters and latent variables, the algorithm finds a set of optimal parameters of the interrelated variables, which can then be solved in detail using the information obtained from the data. Indeed, this is true even for conceptually comparable variables, such as a basic nonhierarchical model with only two parameters and no latent variables.

The extension of the methodology can focus on integrating countervailing machine learning techniques to be a complete defense system in case of attacks that attempt to deceive the models by providing misleading information. Determine strategies and procedures for running the model on specified sets of issues with training and test data generated from the same statistical distribution. Moreover, a future expansion of the proposed system will review the taxonomies of the characteristics of transfer learning, particularly whether and how this system can mitigate them. Finally, learning transfer approaches are investigated from known distribution attack methods seeking to exploit the dynamics of categorization decision-making limits.

## Data Availability

The data used in this study are available from the corresponding author upon request.
